# Antioxidant Vitamins in the Prevention of Atrial Fibrillation: What Is the Evidence?

**DOI:** 10.4061/2011/164078

**Published:** 2011-08-23

**Authors:** Sonia Rasoli, Nicholaos Kakouros, Leanne Harling, Philemon Gukop, Manish Soni, Thanos Athanasiou, Antonios Kourliouros

**Affiliations:** ^1^Department of Cardiothoracic Surgery, St George's Hospital, London SW17 0QT, UK; ^2^Department of Cardiology, Johns Hopkins Hospital, Baltimore, MD 21209, USA; ^3^Department of Cardiothoracic Surgery, Imperial College Healthcare, London W12 0HS, UK; ^4^Department of Cardiac Surgery, St Thomas' Hospital, London SE1 7EH, UK

## Abstract

Atrial fibrillation (AF) is the most common sustained arrhythmia that is associated with significant morbidity and mortality. Current available therapies remain inadequate in symptom control and secondary prevention and are often associated with significant side effects. The mechanisms underlying the pathogenesis of AF are poorly understood, although electrophysiological remodeling has been described as an important initiating step. Recently, increasing evidence implicates oxidative stress and inflammation in the pathogenesis of AF. We searched the literature for evidence to support the use of antioxidant vitamins C and E in the prevention of AF. These vitamins, through their reactive-oxygen-species- (ROS-) scavenging effect, have shown a role in AF prevention in both animal and small clinical studies. The available evidence, however, is currently insufficient to support recommendations for their use in the wider patient population. Larger-scale clinical studies are required to confirm these preliminary results. Research is also required to further the understanding of the processes involved in the pathogenesis of AF and the role of antioxidant therapies to prevent the arrhythmia.

## 1. Introduction

Atrial fibrillation (AF) is the most frequent arrhythmia in the general population and commonest rhythm disturbance after cardiac surgery with a reported incidence of 20% to 50% [1, 2]. According to large epidemiological studies in Europe and in the USA, lifetime risk of developing AF has been estimated to be 1 in 4 for men and women over the age of 40 [3, 4]. The prevalence of AF doubles each decade with advancing age, from 0.5% at the age of 50, to almost 9% at the age of 80–89 years [5]. In a recent UK survey of 4843 people aged over 65, AF was found in 4.7% [6]. Projected data from North America suggests that AF is expected to affect up to 15.9 million people by 2050 [7], and as the observed trend in AF continues to increase, many consider it as the new epidemic [8]. AF is associated with substantial morbidity and mortality as it can lead to thromboembolism and impaired cardiac function. It is also an independent risk factor for death, with a risk ratio of 1.5 for men and 1.9 for women [9]. 

Postoperative AF (POAF) has a peak incidence on the second and third postoperative days [10]. It is often self-limiting as up to 80% of patients return to sinus rhythm by day 3 following correction of electrolyte abnormalities and treatment with *β*-blockers, amiodarone or digoxin. Nonetheless, the onset of POAF may not only significantly prolong hospital stay [11, 12] but may also result in haemodynamic compromise, ventricular arrhythmias, postoperative stroke, myocardial infarction (MI), and heart failure.

The currently available drug therapies for AF have major limitations. They are often associated with treatment failure and proarrhythmic complications, and their prognostic benefit is limited. Radiofrequency ablation has recently shown promising results but is an invasive procedure and it is indicated only in selected patients where conventional antiarrhythmics have failed to provide symptomatic control. Recently, increased effort has been made in the understanding of the pathogenesis of AF and the development of novel therapies to prevent AF, some of which aim at the susceptible structural and physiological substrate.

This paper aims to summarise the current evidence surrounding the use of antioxidant vitamins for the primary and secondary prevention of AF. Furthermore, we propose a potential mechanism by which antioxidant vitamins may exhibit this effect, providing useful insights into the oxidative stress hypothesis in AF. 

## 2. Methods

We carried out an extensive MEDLINE and EMBASE search (1966—November 2010) for original articles, reviews, and meta-analyses using MeSH terms: “atrial fibrillation,” “arrhythmias,” “antioxidants,” “vitamin C,” “ascorbic acid,” “vitamin E,” “tocopherol,” and “vitamin A”. All papers selected were in the English language. We also conducted a “related articles” search for articles identified in MEDLINE. Further relevant papers were examined from reference lists of identified articles. Articles were filtered for studies that looked at antioxidant vitamins in the prevention of AF.

## 3. Pathogenesis of Atrial Fibrillation: The Role of Oxidative Stress

AF is thought to result from multiple random reentrant wavelets that propagate within the atria, perpetuating the atrial arrhythmia. It is a progressive disease, as once it is present it results in electrophysiological and structural remodeling that sustains further AF [13, 14]. An important step in the pathogenesis of AF is electrophysiological remodeling, a term that describes changes that affect excitability and electrical activity of cardiac myocytes [13–15]. The atria in AF are characterised by a diminished action potential and shortening of effective refractory period [13]. The mechanism by which electrophysiological remodeling occurs is poorly understood, though evidence increasingly supports the role of oxidative stress as the key mediator in this process [16–19].

Abnormalities in myocyte calcium handling have been implicated as an important step in initiating the process of atrial electrophysiological remodeling. It is thought that AF initiation is the result of calcium overload, whereas persistent or chronic AF is associated with a reduction in myocyte L-type voltage-gated Ca^2+^ current [13]. The exact mechanism by which calcium abnormalities can cause electrophysiological remodeling is unknown, though increased oxidative stress may play a role [13, 20, 21].

Oxidative stress results from excessive reactive oxygen species (ROS), which are chemically reactive, oxygen-containing ions and small molecules. They are involved in pathological processes such as DNA damage, apoptosis, and cellular hypertrophy as well as signal pathway modulation [21]. The transient nature of free radicals and complexity of available techniques make their measurement in humans difficult. Consequently, a number of surrogate markers are used to assess levels of oxidative stress. Peroxynitrite is a free radical that leads to oxidation of cellular lipids, proteins, and DNA, which can therefore affect protein and enzyme structure and function and cell death [22]. In a study by Mihm and colleagues, myofibrillar proteins were investigated for evidence of oxidative posttranslational protein modification [18]. The excised atrial appendages of patients with AF had increased levels of nitrotyrosine and protein carbonyls, markers of peroxynitrite and hydroxyl radical protein interaction, respectively. In addition, there was evidence of a direct interaction of peroxynitrite with creatinine kinase, causing oxidative damage associated with atrial myofibrillar energetic impairment [18]. Carnes et al. used atrial burst pacing to produce a canine model of atrial fibrillation and confirmed increased levels of protein nitration (suggesting the presence of peroxynitrite anions), increased plasma free radicals, and decreased tissue levels of ascorbate in this model. Furthermore, oral supplementation of ascorbate was shown to attenuate these changes [15].

The pathophysiological link between oxidative stress and AF is unclear. It has been postulated that derangement of ion channel regulatory mechanisms by oxidative stress may be responsible. Activity of the sodium channel is particularly linked with the development of AF [23], and the slowly inactivating sodium currents are reduced by oxidative stress [24]. Peroxide, produced by NADPH oxidase, may increase the amplitude of *K*_v1.5_ channel currents, affecting the action potential repolarisation phase, and hence lead to AF initiation or perpetuation [25–27]. Finally, reduced functionality of L-type calcium channels in AF may help perpetuate the arrhythmia; the reversal of this effect by statins is thought to be a function of their antioxidant activity [28].

## 4. Oxidative Stress in the Pathogenesis of Postoperative Atrial Fibrillation

Cardiac surgery is characterised by acute ischaemic and reperfusion injury, which leads to the release of ROS, causing oxidative stress and a systemic inflammatory response [29]. Although ischaemia/reperfusion plays an important role, some other factors may also contribute. For example, extracorporeal circulation activates inflammatory cells and increases oxidative stress, though this deleterious effect appears abrogated by off-pump cardiac surgery techniques [30–33]. Moreover, pericardial injury and inflammation after cardiac surgery may directly increase atrial oxidative stress, and it is related to atrial arrhythmias [34]. In an experimental study using electron-spin-resonance techniques, reperfusion was shown to generate free radicals, an effect attenuated by antioxidant scavengers [35]. In addition, a persistent increase in plasma lipid peroxidation levels and cardiac release of oxidised glutathione (a marker of oxidative stress) has been demonstrated for 24 hours following aortic cross-clamp release after coronary artery bypass grafting (CABG) [36]. Similarly, Milei and colleagues also showed that following CABG surgery patients had increased cardiac release of glutathione and reduced myocardial levels of antioxidant markers such as vitamin E and ubiquinol [37]. These studies suggest the development of oxidative stress in cardiac surgery, which may account for the high rate of POAF. 

## 5. Antioxidant Vitamins and Atrial Fibrillation Prevention

Normally, cells protect themselves from ROS damage with antioxidant substances including enzymes such as superoxide dismutases, catalases, glutathione peroxidases, and peroxiredoxins. Cells also contain small antioxidant molecules including vitamin C (ascorbic acid), vitamin E (tocopherol), uric acid, and reduced glutathione, which play a vital role in cell protection from oxidant injury. Vitamin C and vitamin E are essential antioxidants and, as such, deserve particular consideration. 

Both vitamin C and vitamin E act by reducing ROS [19]. Vitamin C has been shown to be a major intracellular antagonist of peroxynitrite [38, 39], and it is thought that vitamin E works synergistically with vitamin C. Once alpha-tocopherol is oxidised into alpha-tocopheroxyl radical, it requires an electron donor, such as vitamin C to reduce it, so it can once again function as an antioxidant [40]. Therefore, vitamin C through its direct radical scavenger action and its ability to recycle vitamin E reducing potential is a very effective antioxidant. A summary of the experimental and clinical evidence for the use of antioxidant vitamins in AF prophylaxis is shown in Tables 1 and 2, respectively.

## 6. Vitamin C and Atrial Fibrillation: Experimental Studies

A number of studies have examined the role of antioxidant therapies in the prevention of AF with a particular focus on the atrial tissue surrounding the pulmonary veins (PVs), identified as a source of spontaneous premature depolarisations that trigger and sustain AF [48, 49]. In a recent study, Lin and colleagues investigated the electrophysiological effects of oxidative stress on PVs and the ability of ascorbic acid to attenuate these changes [41]. Hydrogen peroxide, a known ROS, induced burst firing and increased spontaneous activity in PV tissue. These changes were suppressed by pretreatment of PV tissue with ascorbic acid, suggesting oxidative stress has direct physiological effects on PVs, which are attenuated by ascorbic acid.

In their canine model of AF, Carnes and colleagues demonstrated a shortening of the atrial effective refractory period (ERP) with rapid atrial pacing. Treatment with ascorbate attenuated the pacing-induced atrial ERP shortening following 24 to 48 hours of pacing. This is in keeping with the established link between ERP changes and early development of AF [15]. In their study, Shiroshita-Takeshita et al. compared the effect of vitamin C and E on atrial remodeling to that of simvastatin [28]. Dogs were fitted with internal atrial pacing and were subjected to 1 week of atrial tachypacing. Their results are in contrast to those of Carnes et al. as vitamin C and E had no effect on ERP and did not prevent AF, whereas simvastatin both prevented AF and abrogated the ERP-shortening effect. The precise mechanism by which statins prevent AF is not well known, although it is thought to be a consequence of both anti-inflammatory and antioxidant effects [50, 51].

## 7. Prevention of Postoperative AF

Carnes and coworkers examined the effect of ascorbate supplementation on the incidence of postoperative AF in patients undergoing CABG surgery [15]. Oral ascorbic acid was given to 43 patients the night before and for 5 days after CABG surgery. The incidence of POAF in the ascorbate supplementation group was significantly lower than in control subjects (16.3% versus 34.9%, resp., *P* = 0.048). However, it is worth noting that not all risk factors were adequately matched between groups with a higher incidence of diabetes, hypertension, and prior AF in the control group. On the whole, these results support the hypothesis that antioxidant treatment protects against electrophysiological remodeling in AF and reduces incidence of postoperative AF. 

The use of *β*-blocker therapy as standard prophylaxis for the prevention of POAF in cardiac surgery is advocated by evidence-based guidelines (NICE guidelines, American College of Cardiology (ACC)/American Heart Association (AHA)/European Society of Cardiology (ESC)) [52]. The efficacy of ascorbic acid as an adjunct to such *β*-blocker therapy was evaluated in a prospective, randomised trial [45]. One hundred patients over the age of 50 undergoing CABG surgery were assigned to an ascorbic acid group or control group. All patients received *β*-blockers for at least a week prior to surgery and postoperatively. Patients in the ascorbic acid group received 2 g ascorbic acid the night before surgery and 1 g ascorbic acid daily for 5 days postoperatively. Patients in the control group received *β*-blocker therapy alone. Incidence of POAF was 4% in the ascorbic acid group and 26% in the control group (*P* = 0.002). In a similar randomised trial of 170 patients already receiving *β*-blocker therapy, Papoulidis and colleagues studied the effect of ascorbic acid on patients undergoing first-time isolated CABG [12]. The incidence of POAF was significantly lower in the vitamin C group compared to the control (44.7% versus 61.2%, resp., *P* = 0.041). The vitamin C group also had significantly lower intensive care unit stay. Additionally, patients in the vitamin C group who did develop AF had significantly shorter time interval for conversion into sinus rhythm. Vitamin C treatment, therefore, not only has been shown to prevent AF when used alone, but also has synergistic actions when used in combination with standard anti-AF drug treatment [19].

Castillo and colleagues [47] investigated whether antioxidant therapy protocol with combined omega-3 polyunsaturated fatty acids (n-3 PUFAs) and antioxidant vitamins reduces oxidative and inflammatory damage in cardiac tissue. In this study, 95 patients received supplementation with n-3 PUFA or placebo 7 days before on-pump surgery and antioxidant vitamins C and E or placebo from day 2 before surgery until hospital discharge. Atrial tissue was obtained from patients during intervention. Oxidative stress damage in atrial tissue was assessed by measuring the content of reduced glutathione (GSH) and oxidised glutathione (GSSG) ratio (GSH/GSSG), malondialdehyde (MDA) (a marker of lipid peroxidation), and protein carbonylation. Atrial tissue GSH/GSSG ratio was 38.1% higher in supplemented patients compared with placebo (*P* < 0.01). There were also 27.5% lower MDA levels and 24% lower protein carbonylation in the supplemented group compared with placebo (*P* < 0.01). Patients who received PUFA-antioxidant supplementation also showed lower levels of inflammatory biomarkers such as leucocyte count and serum high sensitive CRP. In addition atrial tissue activation levels of NF-kB, a transcriptional messenger central to oxidative stress response, were lower in patients supplemented with antioxidant therapy. NF-kB is a response element of both oxidative stress and inflammatory pathway which is involved in transcription and DNA activation. The evidence from this study supports oxidative and inflammatory-induced damage of cardiac myocytes in ischaemia reperfusion event. Nonetheless, although there was a trend for lower incidence of POAF in the supplemented patient group (22.9% versus 31.9%), this was not statistically significant (*P* = 0.325).

## 8. Vitamins and AF following ST-Elevation Myocardial Infarction (STEMI)

In STEMI, restoration of blood flow has been associated with the release of ROS and reactive nitrogen species by ischaemic tissues, endothelium, and leukocytes. This results in cellular injury and may cause arrhythmia including AF, temporary impairment of systolic function, and endothelial dysfunction. The role of antioxidant vitamins in ischaemic reperfusion injury after therapeutic thrombolysis in patients who had a STEMI was examined by Hicks et al. [46]. Thirty-two patients were randomly assigned to receive adjuvant antioxidant vitamin supplements (vitamin A, vitamin B12, thiamine, vitamin C, vitamin E, cofactors, and minerals), while control group patients were given a placebo. Treatment was administered after the diagnosis of STEMI was established and then at 1, 4, and 12 hours after thrombolytic therapy. The incidence of AF was significantly reduced in patients who received antioxidant vitamins compared to controls (6% versus 44%, *P* < 0.05). The incidence of other arrhythmias such as ventricular premature beats and ventricular tachycardia was also significantly less in the antioxidant group. Furthermore, the neutrophil and total leukocyte count significantly decreased and plasma antioxidant capacity significantly increased 24 hours after reperfusion in patients who received antioxidant therapy. It is notable, however, that 50% of patients in the multi-antioxidant-treated group developed asymptomatic sinus bradycardia, although this did not require treatment or have any identifiable impact on morbidity or mortality. In addition, the simultaneous administration of a combination of vitamins and minerals makes it difficult to ascribe the observed benefit to a single agent. The decrease in AF is postulated to be the result of an increase in plasma antioxidant capacity and consequent neutralisation of ROS by antioxidant vitamins.

## 9. Secondary Prevention of AF with Vitamin C

The concept of utilising non-antiarrhythmic medications for the prevention of AF recurrence following electrical cardioversion, chemical cardioversion, and AF ablation has recently become topical following some encouraging results with statins, angiotensin-converting enzyme inhibitors, and angiotensin receptor blockers [50]. Korantzopoulos et al. examined the effect of oral vitamin C on early recurrence rate of AF after electrical cardioversion of persistent AF [44]. Oral vitamin C was given 12 hours before and for 7 days after cardioversion. AF recurred in 4.5% of patients who received vitamin C supplements compared to 36.3% of patients in the control group (*P* = 0.024). Given that early recurrence of AF following cardioversion is thought to be the result of atrial electrical remodeling [14, 53], this supports the role of vitamin C in protecting against recurrence of AF by attenuating this process [44]. In addition, this study demonstrated that serum inflammatory indices, such as CRP, fibrinogen, and white blood cell count were also significantly reduced in patients supplemented with vitamin C. 

## 10. Vitamin E in AF Prevention

In a study by Sisto and colleagues, 37 patients undergoing CABG surgery received oral vitamin E for 28 days prior to elective surgery or for 2 days prior to urgent surgery for patients presenting with unstable disease [42]. Both patient groups also received vitamin C and allopurinol for two days prior to surgery and for one day after surgery. A further 44 patients were recruited as controls. Although vitamin E supplementation failed to maintain postoperative plasma vitamin E levels in the unstable patients, this group had reduced incidence of arrhythmias (12% versus 42%, *P* = 0.05). Furthermore, relative to the control group, both treatment groups had significantly fewer electrocardiographic ischaemic events and lower incidence of perioperative MI. An important limitation is that the incidence of arrhythmias in this study was reported collectively, making it difficult to get an accurate representation of incidence of AF alone. Additionally, due to the use of combination therapy (vitamin C, vitamin E, and allopurinol), it is difficult to ascribe the favourable outcome to a single agent.

A study by Lassnigg et al. assessed the effect of vitamin E infusions in patients undergoing CABG or valve surgery given for 12–16 hours before and for 48 hours postoperatively [43]. As plasma ROS is difficult to measure directly, malondialdehyde levels (a secondary reactive product) and plasma depletion of vitamin C and E were measured as an indication of oxidative stress. Although vitamin E supplementation maintained plasma vitamin E concentrations, it did not have an effect on the increase in malondialdehyde or the decrease in plasma antioxidant status, which was assessed by measuring plasma bilirubin and uric acid levels. In addition, infusion of vitamin E did not prevent the decrease in plasma vitamin C concentration. The authors question the ability of vitamin E supplementation, without adjuvant vitamin C, in blocking the formation of ROS. This is in accord with the earlier mentioned observation that vitamin E requires electron donation by vitamin C for regeneration of its antioxidant capacity after a single interaction with a ROS.

## 11. Other Antioxidants in AF Prevention

In addition to antioxidant vitamins, some other antioxidant interventions such as N-acetylcysteine (NAC) [54] and nitric oxide [55] have shown promising clinical benefits in reducing oxidative stress and postoperative AF. NAC, a glutathione precursor, is an antioxidant agent that reduces cellular oxidative damage. NAC has been shown to increase L-type calcium channel current and reverse AF-induced remodeling of ion channels [56]. The effect of NAC in the prevention of POAF was evaluated in a prospective, double-blind placebo-controlled trial of 115 patients undergoing CABG and/or valve surgery [54]. The incidence of POAF was lower in the NAC group (5.2%) compared with the placebo group (21.1%) (*P* = 0.019). Nitric oxide, an indirect antioxidant, has also shown to be beneficial in reducing incidence of POAF. Cavolli et al. [55] evaluated the effect of sodium nitroprusside (SNP), a nitric oxide donor, in the prevention of POAF in 100 patients undergoing CABG, with no previous history of AF. The incidence of POAF was 12% in the SNP group and 27% in the control group (*P* < 0.005). In addition, patients treated with SNP showed significantly shorter duration of POAF (5.33 ± 1.86 and 7.55 ± 1.94 h, resp.; *P* = 0.023). Other more potent antioxidants also merit consideration such as astaxanthin, the prototype oxygenated carotenoid or the membrane-permeable scavenger tempol [57]. The effectiveness of these agents remains to be evaluated in further larger studies.

## 12. Challenges and Perspectives of Antioxidant Agents in AF Prevention

Clinical and experimental studies in the antioxidant and, possibly, the antiarrhythmic role of vitamins have provided some useful insights. However, a paradigm shift in this approach to AF prevention may be in store. Using vitamins or other antioxidants to neutralise the already formed intracellular ROS may be trying to lock the door after the horse has bolted. Therapy may, in fact, be better targeted at preventing ROS formation. Studies into the mechanisms of myocardial ROS formation are paving the way for this therapeutic approach. A study by Kim et al. focused on the source of superoxide generation in atrial appendages excised during cardiac surgery from patients with AF compared to control. They found increased activity of NADPH oxidase-2 in AF patients and significant superoxide production by nitric oxide synthases (NOS), suggesting “uncoupling” of this enzyme in AF leading to mitochondrial ROS formation [27]. Although this “uncoupling” may, itself, be a consequence of prior initiating oxidative stress, it provides a novel therapy target. Kim et al. demonstrated reduced basal levels of superoxide in the homogenates of atria from AF patients treated with the mitochondrial inhibitor rotenone. Tetrahydrobiopterin (BH4), a NOS cofactor, can help “recouple” NOS and prevent ROS formation. In a recent abstract of their work, Nishijima et al. report that BH4 reduced inducibility of atrial fibrillation and increased atrial ERP in a tachypacing canine model of heart failure, raising hopes for the “NOS recoupling” approach to AF prevention [58]. 

## 13. Conclusions 

Increasing evidence links oxidative stress with the pathogenesis of electrophysiological and structural remodeling processes that perpetuate AF. Most current available therapies exhibit limited efficacy, are associated with considerable side effects, and may confer no prognostic benefit. Evidence from clinical and experimental studies into the use of antioxidant vitamins in AF prevention indicates that antioxidant vitamins have role in risk reduction of AF. The effect, however, is somewhat variable. An explanation as to why we are not seeing a stronger effect may be because both vitamin C and E are weak antioxidants, and vitamin E, unlike stronger antioxidants (e.g., tempol), requires electron donation after a single interaction with a ROS. Furthermore, at usual oral doses, these vitamins have limited capacity to cross the cell membrane and get to where the superoxide is being produced. Future studies should possibly include the assessment of stronger antioxidant agents, such as tempol, and strive to improve assays of *in vivo* oxidative stress status. In addition, the exact mechanism by which oxidative stress leads to atrial remodeling and AF needs to be elucidated further whereas larger-scale clinical studies are required to confirm the benefits of antioxidant vitamins before they can be considered for routine use.

## Figures and Tables

**Table 1:** Summary of experimental evidence for antioxidant vitamins in AF prevention.

Study	Model	Setting	Intervention/groups	Results	Relevance to AF
Carnes et al. 2001 [15]	Instrumented dogs—atrial electrodes	External ATP	Ascorbic acid versus control	Attenuated shortening of ERP	Prevention

Shiroshita Takeshita et al. 2004 [28]	Dogs—atrial electrodes	External ATP	control versus simvastatin versus vitamin C versus vitamin C & E	Vitamin C/E had no effect	No effect

Lin YK et al. 2010 [41]	Isolated rabbit PVs and LA tissues	*In vitro* hydrogen peroxide oxidation treatment	Ascorbic acid versus control	Reduced PV spontaneous activity and abolished burst PV firing	Prevention

ATP: atrial tachypacing; ERP: effective refractory period; LA: left atrium; PV: pulmonary vein.

**Table 2:** Summary of clinical evidence for the use of antioxidant vitamins in AF prevention.

Study	Design	*N*	Setting	Groups	Endpoint	Follow up	Risk reduction
Sisto et al. 1995 [42]	RCT	81	CABG	Vitamin C/vitamin E/allopurinol versus placebo	Arrhythmias (including AF)	5 days postoperative	30% absolute risk reduction (*P* = 0.05)

Carnes et al. 2001 [15]	Double-blinded, placebo controlled RCT	86	CABG	Ascorbic acid versus control	AF detection	5 days postoperative	18.6% absolute risk reduction (*P* = 0.048).

Lassnigg et al. 2003 [43]	Double-blinded RCT	40	CABG/valve surgery	all-rac-*α*-tocopherol versus placebo	Arrhythmias (including AF)	6 days postoperative	No effect of vitamin E on AF

Korantzopoulos et al. 2005 [44]	Double-blinded RCT	44	DCCV for persistent AF	Vitamin C versus placebo	AF recurrence	1 week post successful DCCV conversion	31.8% absolute risk reduction (*P* = 0.024)

Eslami et al. 2007 [45]	RCT	100	CABG	*β*-blockers + ascorbic acid versus *β*-blockers alone	AF detection	4 days postoperative	22% relative risk reduction (*P* = 0.002)

Hicks et al. 2007 [46]	Double-blinded RCT	32	MI—for thrombolysis	Antioxidant vitamins (A, C, B complex, vitamin E) versus placebo	New-onset AF	2 hours post thrombolysis	38% absolute risk reduction (*P* < 0.05)

Papoulidis et al. 2010 [12]	RCT	170	CABG	Vitamin C versus placebo	AF and rhythm restoration from AF	5 days postoperative	16.5% absolute risk reduction (*P* = 0.04) Reduced ICU stay (*P* = 0.05), reduced hospitalisation time (*P* = 0.04) and reduced time needed for conversion into SR (*P* = 0.047)

Castillo et al. 2010 [47]	Double-blinded RCT	95	CABG	n-3 PUFA/Vitamin C and E versus placebo	AF detection	Hospital discharge	9% absolute risk reduction (*P* = 0.32)

AF: atrial fibrillation; RCT: randomised controlled trial; CABG: coronary artery bypass graft; DCCV: direct current cardioversion; MI: myocardial infarction; ICU: intensive care unit; SR: sinus rhythm; n-3 PUFA: omega 3 polyunsaturated fatty acids.
